# Enhanced Recovery After Surgery (ERAS) for Esophagectomy: A Paradigm Shift in Perioperative Care

**DOI:** 10.1245/s10434-024-16139-2

**Published:** 2024-09-16

**Authors:** Peter P. Grimminger

**Affiliations:** Department of General, Visceral and Transplantation Surgery, University Medical Centre of the Johannes Gutenberg-University Mainz, Mainz, Germany

The evolving landscape of esophageal cancer treatment highlights surgery as a cornerstone for achieving high cure rates. Despite its curative potential, esophagectomy is a complex procedure marked by extensive esophageal resection and reconstruction, resulting in substantial postoperative morbidity and a significant burden of symptoms. In this context, the Enhanced Recovery After Surgery (ERAS) protocol offers a transformative approach to perioperative care, aimed at reducing surgical stress and symptom burden, thereby promoting rapid recovery and enhancing the quality of life for patients.^1^

The complexity of esophagectomy necessitates an optimal surgical model to mitigate the inherent risks and enhance patient outcomes. ERAS protocols have been increasingly adopted across various surgical disciplines, including esophageal cancer surgery, due to their potential to improve perioperative care. Studies have demonstrated that ERAS protocols are both safe and effective, significantly reducing perioperative complications and enhancing recovery.^2–5^ However, compared with conventional models, heterogeneity in perioperative complications remains, primarily due to the intricate steps involved in esophagectomy, such as lymphadenectomy, esophageal reconstruction, and anastomosis.

The study by Huang and co-authors tackles one of the key controversies in evaluating the effectiveness of ERAS protocols, which is the postoperative length of stay.^6^ While ERAS aims to enhance the overall quality of surgical rehabilitation, not just the speed, there is often a lack of discernible difference in postoperative length of stay when discharge standards are not rigorously compared. This perception limits the wider adoption of ERAS protocols. Another critical innovation within the ERAS framework is the non-tube no fasting protocol, which deliberately avoids traditional elements such as drainage near the anastomosis, nasogastric tubes, nutrition tubes, and abdominal jejunostomy. Instead, it emphasizes early oral feeding, reduced intravenous fluids, and early mobilization. This approach significantly alleviates adverse surgery-related experiences and aligns more closely with the natural physiological recovery process. Despite its benefits and patient preference, its adoption remains limited, primarily due to the perceived equivalence in clinical outcomes with conventional models. To truly appreciate the benefits of ERAS protocols, it is essential to consider patient-reported outcomes that reflect the patient’s perspective on quality of life. The study by Huang and co-authors demonstrates that the non-tube no fasting ERAS protocol not only reduces perioperative complications and shortens postoperative length of stay, but also provides superior patient-reported outcomes within 6 months after surgery. The study showed significant reductions in complications such as pneumonia, arrhythmia, and overall complications in the ERAS group compared with the conventional group. These findings align with other ERAS-related esophagectomy studies.^3–5^ The ERAS protocol’s advantages extend beyond clinical metrics. The study by Huang and co-authors highlights improvements in psychological health, social functioning, and overall global health status within the ERAS group. These benefits are attributed to key components of the protocol that help patients return to their preoperative state more rapidly, facilitating immediate mental relaxation and smoother reintegration into normal life. Notably, the ERAS group showed sustained advantages in role, emotional, cognitive, and social functioning up to 3 months post-surgery.

Despite its challenges, such as the single-center retrospective design, the study provides compelling evidence for the superiority of the non-tube no fasting ERAS protocol in enhancing postoperative recovery and quality of life. Future research should focus on standardizing ERAS pathways across multiple centers and incorporating patient-reported outcomes to explore the key operative components that maintain long-term benefits.

In conclusion, the ERAS protocol for esophagectomy represents a significant advancement in perioperative care. It not only reduces perioperative complications and shortens postoperative length of stay, but also enhances patient-reported outcomes, maintaining overall quality of life. This editorial calls for a renewed focus on patient-centered care, urging esophageal surgeons and healthcare administrators to recognize the full potential of ERAS protocols beyond immediate clinical outcomes. Through collaborative research and continuous refinement, the true advantages of ERAS can be realized, setting a new standard in surgical recovery for patients with esophageal cancer.
